# Efficacy of Shikonin against Esophageal Cancer Cells and its possible mechanisms *in vitro* and *in vivo*: Erratum

**DOI:** 10.7150/jca.92106

**Published:** 2023-11-28

**Authors:** Jian-Cai Tang, Jia Zhao, Feng Long, Jian-ye Chen, Bo Mu, Zhen Jiang, Yonggan Ren, Jian Yang

**Affiliations:** 1 Department of Biochemistry;; 2 School of Pharmacy;; 3 Department of Pharmacy, Nan Chong Central Hospital;; 4 Pathogenic Biology and Immunology Experiment Teaching Center, North of Si Chuan Medical University, China.

In the original version of our article, there was two errors in Fig. 4A. Specifically, the representative images of PI3K and AKT of ECA109 cells in Figure 4A are incorrect. The correct image is provided below. This correction will not affect the results and conclusions. The authors apologize for any inconvenience this may have caused.

## Figures and Tables

**Figure 4A:**
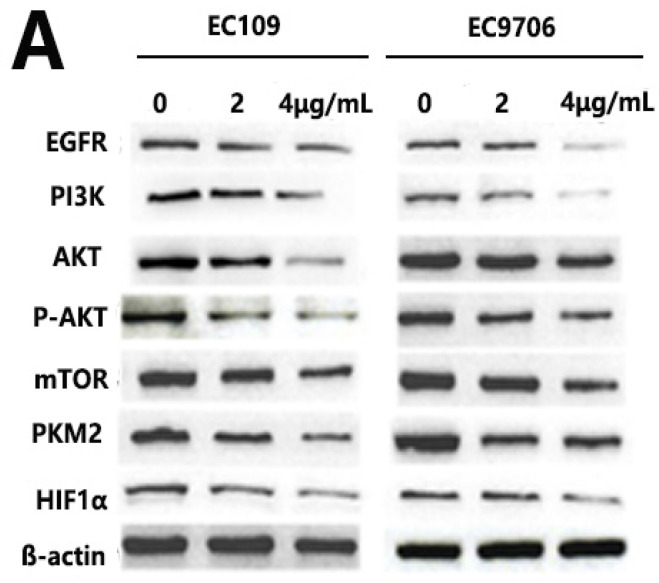
** Shikonin inhibited EGFR/PI3K/AKT signal pathway.** Ec109 and EC9706 cells were treated with shikonin(0,2,4μg/ml) for 24h and the total protein were extracted, then the expression of EGFR, PI3K, AKT, p-AKT, mTOR, PKM2 and HIF1ɑ were examined by Western blot. The results showed that shikonin decreased the expression of EGFR, PI3K, AKT, p-AKT, mTOR, PKM2 and HIF1ɑ.

